# Peer Review of “A Full-Scale Agent-Based Model to Hypothetically Explore the Impact of Lockdown, Social Distancing, and Vaccination During the COVID-19 Pandemic in Lombardy, Italy: Model Development”

**DOI:** 10.2196/32796

**Published:** 2021-09-10

**Keywords:** epidemiology, computational, model, COVID-19, modeling, outbreak, virus, infectious disease, simulation, impact, vaccine, agent-based model

*This is a peer-review report submitted for the paper “A Full-Scale Agent-Based Model to Hypothetically Explore the Impact of Lockdown, Social Distancing, and Vaccination During the COVID-19 Pandemic in Lombardy, Italy: Model Development”.*

## Round 1 Review

### General Comments

The paper [1] describes an agent-based model for investigating the COVID-19 spread in Lombardy. The importance of this study is evident. Additionally, it is interesting work. However, this manuscript needs to be enhanced more before publication. My main concerns are about the points below.

### Specific Comments

#### Major Comments

1. The event of disease spread has been extremely simplified. As you know, the outbreak of a disease is affected by lots of factors.

2. The Introduction lacks enough references to previous research.

3. The model has not been validated and verified, which are the most important tasks in proving the correct performance of the model developed.

4. The movement of all agents has been considered randomly, while in reality, it does not happen in this way.

5. The materials and methods lack information about the way the model was developed. All necessary information about the model needs to be made known—attributes and behaviors of the agents, interactions between the agents, etc.

6. As you know, one of the advantages of the agent-based model approach is its consideration of the geography of the environment and simulation of the exact locations of people and places. The diversity of the population affects the spread of the disease as well as interactions. If the population density remains constant, but people do not have interactions with each other, the disease does not spread.

#### Minor Comments

1. The manuscript needs to be thoroughly proofread by a native English speaker.

2. The Abstract lacks results, which is an important part of the Abstract. Innovations and aims of the manuscript have not been expressed clearly as well as the contributions of the manuscript.

3. Figure 1 does not include any information.

4. The way the manuscript has been written is not appropriate. It has not been developed like a manuscript. It needs to be rewritten.
